# Hereditary transthyretin-mediated amyloidosis with polyneuropathy: baseline anthropometric, demographic and disease characteristics of patients from a reference center

**DOI:** 10.1590/0004-282X-ANP-2020-0590

**Published:** 2021-11-30

**Authors:** Vanessa Cristina Cunha Sequeira, Maria Alice Penetra, Lisa Duarte, Fernanda Reis de Azevedo, Raphael Santa Rosa Sayegh, Roberto Coury Pedrosa, Márcia Waddington Cruz

**Affiliations:** 1 Universidade Federal do Rio de Janeiro, Hospital Universitário Clementino Fraga Filho, Centro de Paramiloidose Antônio Rodrigues de Mello, Rio de Janeiro RJ, Brazil. Universidade Federal do Rio de Janeiro Hospital Universitário Clementino Fraga Filho Centro de Paramiloidose Antônio Rodrigues de Mello Rio de Janeiro RJ Brazil; 2 Alnylam Pharmaceuticals Inc, São Paulo SP, Brazil. Alnylam Pharmaceuticals Inc São Paulo SP Brazil; 3 Universidade Federal do Rio de Janeiro, Hospital Universitário Clementino Fraga Filho, Instituto do Coração Edson Saad, Rio de Janeiro RJ, Brazil. Universidade Federal do Rio de Janeiro Hospital Universitário Clementino Fraga Filho Instituto do Coração Edson Saad Rio de Janeiro RJ Brazil

**Keywords:** Amyloidosis, Amyloid Neuropathies, Familial, Anthropometry, Demography, Therapeutics, Amiloidose, Neuropatias Amiloides Familiares, Antropometria, Demografia, Terapêutica

## Abstract

**Background:**

Hereditary transthyretin-mediated (hATTR) amyloidosis with polyneuropathy is a rare, inherited, multisystem, and often fatal disease caused by a variant in transthyretin (TTR) gene. Baseline characteristics of patients, especially anthropometric data, are scarce in the literature, and they are relevant to define effective treatment strategies.

**Objective:**

This study aimed to describe baseline demographic, anthropometric, and disease characteristics in a cohort of patients from a reference center in Brazil.

**Methods:**

Symptomatic patients not previously included in clinical trials and eligible for treatment were enrolled. Ethnicity, state of residence, age, sex, weight, height, body mass index (BMI), TTR variant, and Polyneuropathy Disability Score (PND) at diagnosis were analyzed.

**Results:**

Among the 108 patients enrolled, 58.33% were male, 60.19% were Caucasian, and 83.33% lived in the Southeast region. Mean age was 51.61 (±16.37) years, mean weight was 65.76 (±15.16) kg, mean height was 168.33 (±10.26) cm, and mean BMI was 23.11 (±4.45) kg/m^2^. The most prevalent variant was V30M (86.11%). Patients with PND score 0 presenting autonomic neuropathy were 14.81%. Patients with PND score I-II and III-IV were 52.78 and 32.41%, respectively. Mean weight and BMI were significantly lower in patients with sensory-motor manifestations.

**Conclusions:**

This is the largest cohort of patients in Brazil for whom anthropometric characteristics have been described. Baseline demographic, anthropometric, and disease data indicate that delay in diagnosis of hATTR amyloidosis with polyneuropathy is still a problem and that efforts must be made to expedite diagnosis and maximize opportunities for new disease-modifying treatments.

## RESUMO

**Antecedentes:**

Amiloidose hereditária por transtirretina (hATTR) com polineuropatia é uma doença rara, hereditária, multissistêmica, frequentemente fatal, causada por mutação no gene da transtirretina (TTR). Características dos pacientes ao diagnóstico, especialmente dados antropométricos, são raros na literatura, e são relevantes para definir estratégias terapêuticas eficazes.

**Objetivo:**

Este estudo objetivou descrever características demográficas, antropométricas, e da doença, numa coorte de pacientes de um centro de referência no Brasil.

**Métodos:**

Pacientes sintomáticos, não incluídos previamente em ensaios clínicos e elegíveis para tratamento foram recrutados. Etnia, estado de residência, idade, sexo, peso, altura, índice de massa corporal (IMC), mutação TTR e *Polyneuropathy Disability Score* (PND) ao diagnóstico foram analisados.

**Resultados:**

Foram incluídos 108 pacientes, sendo 58,33% do sexo masculino e 60,19% caucasianos. Na região sudeste residem 83,33%. A idade média foi 51,61 (±16,37) anos. O peso médio foi 65,76 (±15,16) Kg, a altura média foi 168,33 (±10,26) cm e o IMC médio foi 23,11 (±4,45) Kg/m^2^. A mutação mais prevalente foi V30M (86,11%). Pacientes com escore PND 0 apresentando neuropatia autonômica foram 14,81%. Pacientes PND I-II e PND III-IV totalizaram 52,78% e 32,41%, respectivamente. Peso e IMC médios foram significativamente menores em pacientes com manifestações sensitivo-motoras.

**Conclusões:**

Esta é a maior coorte de pacientes do Brasil que teve dados antropométricos descritos. Dados basais demográficos, antropométricos e das manifestações da doença indicam que o atraso no diagnóstico ainda é um problema, e esforços precisam ser feitos para acelerá-lo a fim de maximizar as oportunidades com os novos medicamentos que alteram o curso da doença.

## INTRODUCTION

Amyloidosis comprises a heterogeneous group of diseases that is fundamentally characterized by the deposit of insoluble amyloid fibril in several tissues and organs, causing progressive and severe multisystem clinical manifestations^1^^-^^4^. 

Transthyretin-mediated (ATTR) amyloidosis is caused by the extracellular deposit of transthyretin (TTR), a soluble tetrameric protein produced mainly in the liver, but also in the choroid plexus in the brain and in the retinal pigment epithelium, which carries retinol and thyroxin in blood and cerebrospinal fluid. Two ATTR amyloidosis subtypes are described, based on the pathophysiology and the type of amyloid fibrils that deposit in tissues: the wild-type ATTR (ATTRwt) amyloidosis, caused by the deposit of native transthyretin, and the hereditary ATTR (hATTR) amyloidosis, also known as ATTRv (v for variant) amyloidosis, caused by the deposit of both variant and wild-type transthyretin. Hereditary ATTR is an autosomal dominant disease caused by a variant in TTR gene. The variant TTR tetramer becomes unstable and then dissociates into monomers, which suffer a misfolding, aggregate, and finally accumulate as amyloid fibrils in the tissues, mainly in the nervous system, but also in heart, kidney, and gastrointestinal tract^1^^-^^5^. 

More than 120 pathological variants of TTR gene have already been identified, V30M being the most prevalent worldwide^6^^-^^8^. The variants influence many aspects of the disease, such as clinical presentation, severity, and overall survival. Some variants, such as V30M, cause mainly neurological manifestations (hATTR amyloidosis with polyneuropathy), while other variants affect mainly the heart (hATTR amyloidosis with cardiomyopathy). However, it is common that neurological and cardiac manifestations coexist in a single patient, leading to a mixed phenotype disease. According to the age of signs and symptoms onset, patients are classified as having early onset (<50 years old) or late onset (> 50 years old) disease^4^^,^^6^^-^^11^. Variants related mainly to cardiomyopathy are associated to a lower overall survival^11^^-^^13^. 

Hereditary transthyretin-mediated amyloidosis with polyneuropathy (formerly known as familial amyloidotic polyneuropathy) was initially described by Corino de Andrade in 1952, after his observations of patients with peculiar neurological manifestations in Póvoa do Varzim, Portugal^14^. It is an insidious, progressive disease that affects the autonomic and sensory-motor nervous systems. According to Coutinho et al.^5^, three evolving stages of sensory-motor polyneuropathy are identified: In stage 1, typically mild sensory manifestations start in the lower limbs, and unassisted walking is preserved; in stage 2, the patient needs assistance for walking due to the progressive weakness that affects muscles of the lower limbs; in stage 3, sensory manifestations are severe, and due to the severe weakness or flaccid paralysis of all limbs, the patient is wheelchair-bound or bedridden^4^^,^^15^. In clinical practice, the polyneuropathy disability (PND) score is commonly used to stage the degree of sensory-motor impairment. Patients at PND 0 have no impairment; at PND I, sensory disturbances are observed but the ability to walk is maintained; at PND II, the ability to walk is impaired, but the patient is able to walk without a cane or crutch; at PND IIIa, the patient requires a cane or crutch to walk; at PND IIIb, the patient requires two canes or crutches to walk; and at PND IV, patient is wheelchair-bound or bedridden^4^.

Autonomic neuropathy very often occurs together with sensory-motor neuropathy and can even precede it in disease presentation. Many organs and systems can be affected by autonomic neuropathy, leading to potentially life-threatening situations, such as cardiac arrhythmia and uncontrolled arterial blood pressure^10^^,^^16^. After approximately 10 to 15 years, patients usually die due to cachexia, severe infections, and cardiac complications^8^^,^^17^. Clinical manifestations of polyneuropathy and direct involvement of other organs by hATTR amyloidosis are summarized in Table 1.

**Table 1.** Clinical manifestations of hATTR amyloidosis^1^^,^^18^.

Organ / system	Clinical manifestations
Sensory-motor nervous	Neuropathic pain, altered sensation, numbness and tingling, muscle weakness, impaired balance, difficult walking
Autonomic nervous	Orthostatic hypotension, recurrent urinary tract infection (due to urinary retention), sexual dysfunction, sweating abnormalities
Gastrointestinal	Nausea and vomiting, changes in motility (i.e., diarrhea, constipation, gastroparesis, early satiety), unintentional weight loss
Cardiovascular	Conduction blocks, cardiomyopathy, palpitations and arrhythmia, mild valvular regurgitation, shortness of breath, edema
Ocular	Vitreous opacification, glaucoma, abnormal conjunctival vessels, pupillary abnormalities
Renal	Proteinuria, renal failure
Musculoskeletal	Carpal tunnel syndrome
Central nervous	Progressive dementia, headache, ataxia, seizures, spastic paresis, stroke-like episodes

Initially, this disease was thought to be confined to a few countries such as Portugal, Sweden, Japan, and Brazil, but it is now known to be prevalent worldwide. The exact prevalence is still unknown, but it is estimated to affect around 50,000 people worldwide, being a rare disease^7^^,^^17^. In Brazil, it is estimated that there are more than 5,000 patients with hATTR amyloidosis with polyneuropathy^19^. 

Several more common diseases associated with neuropathy are part of the differential diagnosis, including diabetes, chronic inflammatory demyelinating polyneuropathy (CIDP), toxic neuropathies, Fabry disease, Charcot-Marie-Tooth disease, autonomic and sensory hereditary neuropathies, and light chain amyloidosis. In Brazil, leprosy with neurological manifestations is also an important differential diagnosis due to its prevalence. After clinical suspicion, the final diagnosis goes through confirmatory tools that include histopathology and genetic testing. The latter are also important to establish the correlation between the variant and the expected clinical evolution and to detect early asymptomatic carriers of the TTR gene variant ^20^^,^^21^.

Orthotropic liver transplantation (OLT) was the first disease-modifying treatment for hATTR amyloidosis with polyneuropathy. The aim of OLT is to cease the production of mutant TTR protein, since it is almost totally produced (~98%) in the affected liver. This is a complex and expensive treatment that does not reverse existing amyloid deposits nor does it have the same efficacy in all variants. Thus, this treatment does not address properly all important aspects of the disease^4^^,^^20^^,^^21^. In the last decade, disease-modifying drugs were approved for the treatment of hATTR amyloidosis with polyneuropathy by regulatory agencies worldwide, including Brazil^22^^-^^24^. These drugs have distinct mechanisms of action that address different steps of the pathophysiology of hATTR amyloidosis with polyneuropathy. The route of administration, posology, and indication within the clinical spectrum of the disease also vary for these drugs^25^^-^^28^. Such facts call for a deeper knowledge of the clinical, demographic, and anthropometric characteristics of patients with hATTR amyloidosis with polyneuropathy, allowing the health care system to develop a better approach for these patients considering the currently available treatment options and their potential impact on disease prognosis. 

The aim of this study was to describe the baseline demographic, anthropometric, and disease characteristics of untreated patients with hATTR amyloidosis with polyneuropathy from a reference center in Rio de Janeiro, Brazil. 

## METHODS

### Study design

This was a cross-sectional, non-interventional study, carried out at CEPARM, a reference center for amyloidosis located at Federal University of Rio de Janeiro, Brazil.

### Inclusion criteria

The inclusion criteria were symptomatic patients, eligible for treatment, who were not previously included in any other study or registry about the disease.

### Data

Baseline demographic (ethnicity, age at diagnosis, region of residence), anthropometric (weight, height and body mass index -BMI) and disease (TTR gene variant and Polyneuropathy Disability Score -PND) characteristics were collected from patients’ medical records and analyzed.

### Statistical analysis

Categorical variables were described by frequency and distribution. For continuous variables, mean and standard deviation (SD) values were described. To analyze if mean age was higher and mean weight and BMI lower in PND I-IV patients in comparison with PND 0 patients at diagnosis, one-tailed t-tests were applied, assuming p-values <0.05 as statistically significant.

## RESULTS

Among all patients treated in this reference center, 108 met the inclusion criteria. 

**Figure 1.**
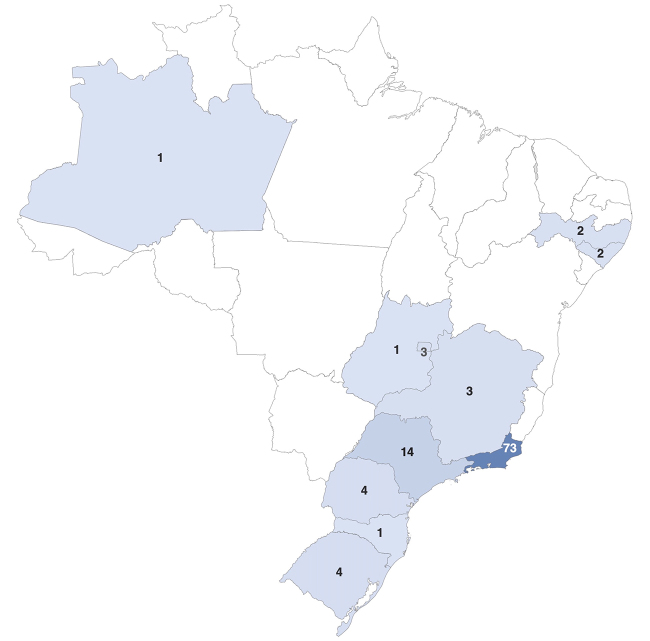
Distribution of patients (n) treated at CEPARM per state of residence.

Regarding demographic data, 58.33% of all patients enrolled in the study were male. Ethnic analysis showed that 60.19% were Caucasians. Most of the patients (83.33%) lived in the Southeast region of Brazil and the remaining 16.67% were distributed in all other regions. The distribution of patients by state of residence is outlined in Figure 1. The mean age at diagnosis was 51.61 (±16.37) years.

**Figure 2.**
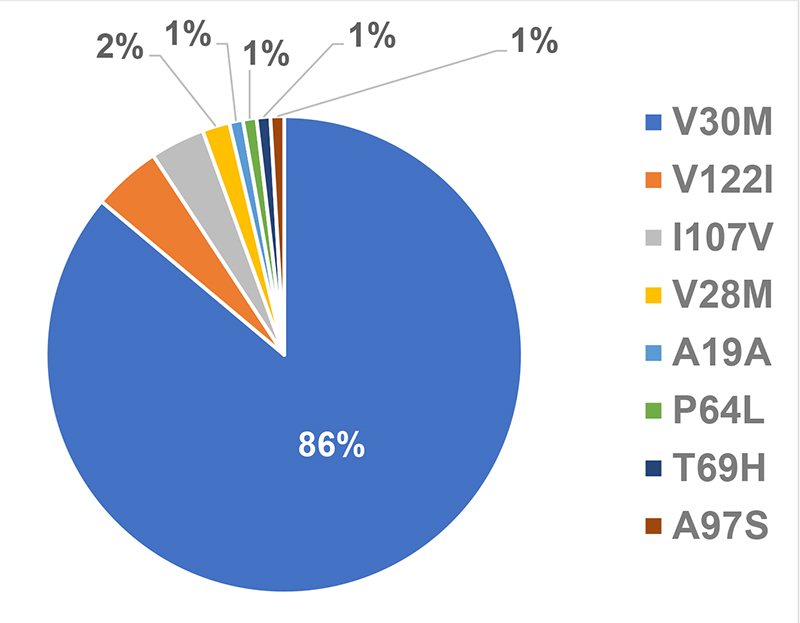
Distribution of TTR variants.

The most prevalent TTR variant was V30M, which affected 86.11% of the patients. The frequency of each variant found is detailed in Figure 2. 

**Figure 3.**
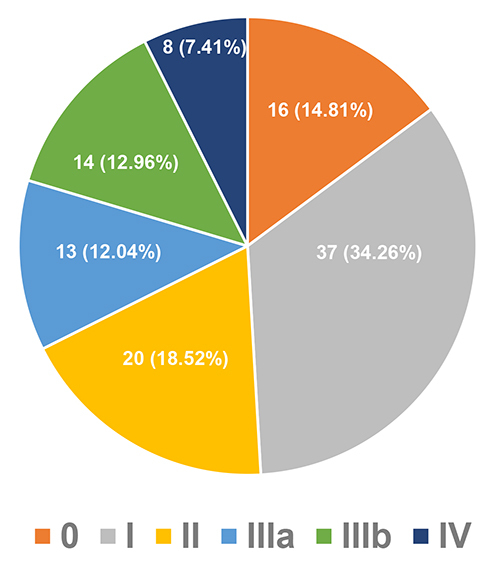
Distribution of patients (n, %) according to PND scores at diagnosis.

Patients diagnosed at PND score 0 but already had autonomic neuropathy were 14.81%, while patients with sensory-motor manifestations at diagnosis accounted for 85.19% (52.78% with PND I-II and 32.41% with PND III-IV). The distribution of patients according to PND score at diagnosis is shown in Figure 3.

The mean weight was 65.76 (±15.16) kg, the mean height was 168.33 (±10.26) cm, and the mean BMI was 23.11 (±4.45) kg/m^2^. 

**Figure 4.**
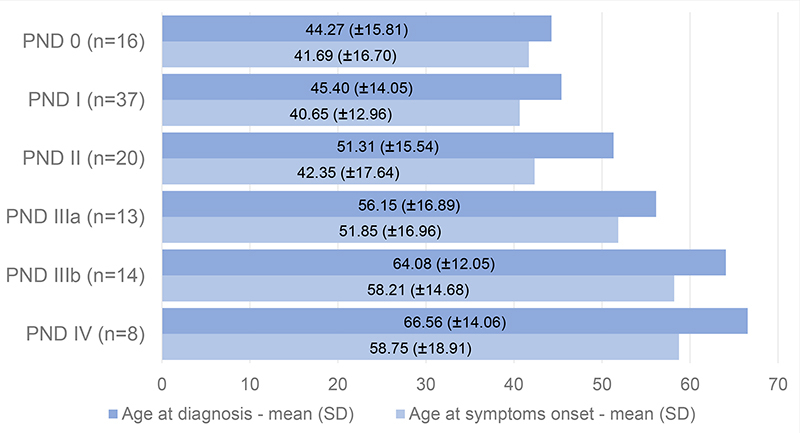
Ages according to each PND score at diagnosis and symptoms onset.

**Figure 5.**
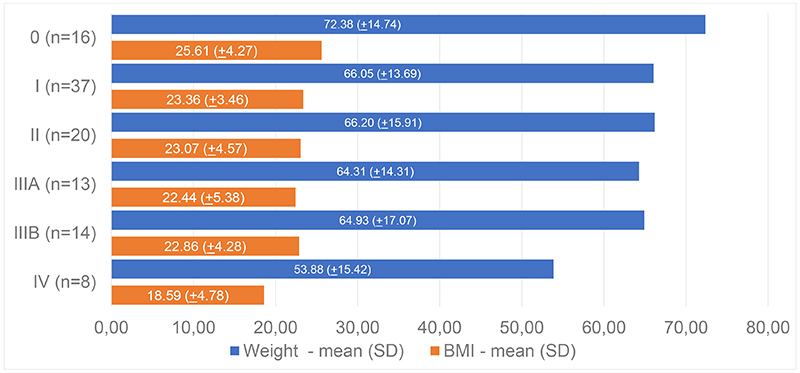
Weight and BMI according to each PND score at diagnosis.

Mean ages at diagnosis and at symptoms onset, weight, and BMI related to each PND score at diagnosis are shown in Figures 4 and 5, respectively. The differences in mean age at diagnosis, weight, and BMI between PND 0 and PND I-IV patients are shown in Table 2.

**Table 2.** Differences in mean age, weight and BMI between PND 0 and PND I-IV at diagnosis.

	PND 0 n=16	PND I-IV n=92	p-value
Age, years mean (SD)	44.27(±15.81)	52.89 (±16.21)	< 0.03
Weight, kg mean (SD)	72.38 (±14.74)	64.61 (±15.02)	< 0.03
BMI, kg/m^2^ mean (SD)	25.61 (±4.27)	22.68 (±4.36)	< 0.01

## DISCUSSION

Since its first description, hATTR amyloidosis with polyneuropathy has posed challenges to physicians not only in relation to its diagnosis, but also in relation to its effective treatment. The very low prevalence and the differential diagnosis with more prevalent diseases associated with polyneuropathy are concurrent factors that lead to misdiagnoses, which prolongs the patient’s journey to a definitive diagnosis. Recently, Cruz et al. reported that 35.6% of patients from a cohort at this center received a misdiagnosis before a definitive diagnosis, the most common of which was CIDP (16.7%). The median times for diagnosis for men and women since the onset of signs and symptoms were 2.6 and 5.0 years, respectively^19^. It is estimated that the patient’s journey to diagnosis takes 4 years on average and that 3 to 4 physicians are consulted in this period^20^^,^^29^. In Brazil, the estimated mean time to diagnosis is 5.9 years^21^. 

The establishment of well-structured reference centers that provide a multidisciplinary approach by professionals familiar with the disease aims to shorten the patient’s journey to diagnosis and to carry out the best disease management strategy for each individual. In Brazil, there are few centers with significant experience in hATTR amyloidosis with polyneuropathy^21^. Maybe this situation helps to explain the fact that CEPARM takes care of patients from states far from its location. Since the establishment of this reference center, the number of patients with hATTR amyloidosis diagnosed and treated there has increased, suggesting that awareness of the disease is probably increasing^19^. However, the differences between the mean ages at symptoms onset and at diagnosis according to PND scores found in this study confirm the long patient journey to the diagnosis. Actually, two cohorts of Brazilian patients in whom the V30M variant was the most prevalent (91.6% -100%) had median ages for the beginning of clinical manifestations between 32 -32.5 years^30^^,^^31^. The significantly higher mean age at diagnosis found in patients with scores PND I -IV when compared to PND 0 also confirms the delayed diagnosis, which exposed patients to neurological deterioration for a longer period. The way to accelerate diagnosis and avoid advanced neurological deterioration is probably to recognize in a timely manner key clinical manifestations of autonomic neuropathy, which may manifest earlier in the disease, as already mentioned. Efforts should be made to raise awareness of key autonomic signs and symptoms in frontline medical specialties.

Anthropometric data of patients with hATTR amyloidosis with polyneuropathy are scarce in the literature, making comparative analysis across different populations difficult. This is the largest cohort of Brazilian patients whose anthropometric data have been described. In this cohort, mean weight and mean BMI showed a significant decrease between PND 0 and PND I-IV, demonstrating the expected correlation with neurological deterioration, although BMI always remained within the normal range across all PND scores. According to data collected from 1,114 patients from Transthyretin Amyloidosis Outcome Survey (THAOS) registry, unintentional weight loss is the most common gastrointestinal manifestation in hATTR amyloidosis with polyneuropathy. It affects 31.5% of the patients and remains the most prevalent sign at all stages of the disease. Its multifactorial pathophysiological mechanism is not completely elucidated yet, but the early satiety and the increasing body metabolism caused by the inflammation in response to amyloid accumulation in tissues seem to be part of these mechanisms. It is worth noting that unintentional weight loss can start before other clinical gastrointestinal manifestations. Gastrointestinal symptoms were negatively associated with patients' nutritional status and health related quality of life (HRQoL)^10^.

It is well known that the amount of accumulated amyloid fibrils in tissues correlates with the severity of the disease^32^^,^^33^. Late diagnosis not only exposes the patient to a higher amyloid deposition in tissues leading to a more severe disease, but also means a loss of opportunity regarding new disease-modifying drugs for hATTR with polyneuropathy that are currently available. According to the mechanisms of action proposed, their benefits result from a reduction or halt of new amyloid deposits in tissues and organs. Therefore, the more advanced the disease at the beginning of treatment, the less benefits patients receive from these new drugs^25^. Three drugs are currently approved in Brazil for hATTR amyloidosis with polyneuropathy: inotersen and patisiran are approved for patients with disease stages 1 and 2 and tafamidis is approved for patients with initial or intermediate disease stage^26^^-^^28^. The Food and Drug Administration (FDA) has approved inotersen and patisiran for treatment of hATTR amyloidosis with polyneuropathy, regardless its stage, while European Medicines Agency (EMA) has approved both drugs for patients with disease stages 1 and 2^34^^-^^37^. Tafamidis is not approved by the FDA for treatment of hATTR amyloidosis with polyneuropathy, and EMA has approved it only for patients with stage 1 disease^38^^,^^39^. Tafamidis is administered orally once a day, inotersen is a weekly subcutaneous injection, and patisiran is administered intravenously and its dosage is based on the patient’s body weight^26^^-^^28^. Knowledge of patient anthropometric and demographic characteristics and disease manifestations is important for comprehensive treatment strategies. 

Studies are currently under way to assess the efficacy of drugs designed to dissolve amyloid deposits in tissues^25^. This new and promising mechanism of action, together with drugs already shown to be effective, can further improve the individualization of treatment for hATTR amyloidosis with polyneuropathy. Combining drugs with different mechanisms of action aimed at improving treatment efficacy may become a reality in the future^7^^,^^25^. 

In conclusion, reference centers for hATTR amyloidosis with polyneuropathy play an important role in providing a better and more comprehensive approach to the patient. Despite a higher awareness of the disease, baseline demographic, anthropometric, and neurological data indicate a significant number of patients are diagnosed late in Brazil. Current and future disease-modifying treatments, together with a deeper knowledge of patients’ characteristics in treatment centers, will leverage a continuous search for better treatment strategies. This is the largest cohort of patients in Brazil that had their anthropometric and demographic data described. More studies like this are encouraged to support more efficient disease management by the health care system.
